# Comparison of personality differences of Polish mountaineers

**DOI:** 10.3389/fpsyg.2023.1120238

**Published:** 2023-02-22

**Authors:** Paweł Piepiora, Justyna Bagińska, Kazimierz Witkowski, Justyna Nakonieczna, Zbigniew Piepiora

**Affiliations:** ^1^Faculty of Physical Education and Sports, Wroclaw University of Health and Sport Sciences, Wrocław, Poland; ^2^Wroclaw Business University of Applied Sciences, Wrocław, Poland; ^3^Faculty of Environmental Engineering and Geodesy, Wrocław University of Environmental and Life Sciences, Wrocław, Poland

**Keywords:** sports psychology, Big Five, mountaineering, Alpine climbing, Himalayan climbing, innovative agonology

## Abstract

A noticeably increased interest in mountain climbing, both as the form of extreme sport and a form of tourism, has been observed in Poland recently. The assumption of this study is that practicing different varieties of mountaineering influences the personality of Polish climbers in a different manner. The aim of the research was to compare the personality differences of Polish mountaineers. To this aim, the population of Polish high-performance mountaineers was studied (*N* = 81; including 39 women and 42 men). Due to the type of mountaineering practiced, the respondents were divided into Alpine climbers (*n* = 48) and Himalayan climbers (*n* = 33). The average age of the surveyed climbers is 33.85 years. The Big Five model was used including the NEO-FFI Personality Questionnaire and the analyzes were performed using the IBM SPSS Statistics statistical method package, version 27.0. Statistically significant differences were noted only for agreeableness *F*(1.77) = 5.05, *p* = 0.027. The Alpine climbers showed a higher level of agreeableness than the Himalayan climbers. After taking into account the Sidak amendment, significant differences in the level of agreeableness were found only among women. Comparisons between other personality traits were not statistically significant. There is a significant difference between the personalities of Polish Alpine climbers and Polish Himalayan climbers in the dimension of agreeableness only among women: female Alpine mountaineers are more agreeable than Himalayan mountaineers. It was presumed that in terms of ethics in the high mountains, the social competences of Alpine mountaineers are much more developed than that of Himalayan mountaineers.

## Introduction

The mountains have fascinated people for centuries. Those living at the foot of the mountains worshiped them. Some of these beliefs have survived to this day and still play an important role in the highlander community (Jeong et al., 2002). Mountains are defined as parts of land located at higher altitudes than the highlands and lowlands, separated from them by natural boundaries. When classifying mountain heights, one takes into account the characteristic altitudinal zonation of fauna and flora, the presence of large differences in relative altitudes or the absolute height above sea level (Wierciński, 2021). In sports theory, it is assumed that the peaks of the low mountains are up to 1,000 m above sea level, the middle mountains are at an altitude of 1,001–2,500 m, and the high mountains are above 2,500 m above sea level (Kosendiak, 2005).

Physical activity in the high mountains is called mountaineering or alpinism. It is understood as climbing difficult climbing routes, walls and ridges at high altitudes (Biswas et al., 2022). In Poland the following basic varieties of high mountain climbing are distinguished, with respect to the terrain difficulties and locations: mountaineering practiced in the Tatra Mountains (over 2,000 m above sea level); Alpine mountaineering (over 4,000 m above sea level); and Himalayan mountaineering (over 7,000 m above sea level) (Kosendiak, 2005). It is an extreme sport in which success depends on proper preparation, appropriate knowledge and experience. An inseparable element of mountaineering is preparation for specific weather conditions and threats coming from the surrounding terrain, and the selection of the necessary equipment (Żoczek et al., 2017) as well as the choice of the appropriate company. The basis for a mountaineering expedition preparation is comprehensive physical training (Prokopczyk and Wochyński, 2022). It is based mainly on endurance and strength components (Prokopczyk and Sokołowski, 2022) and targeted climbing training (Hamid et al., 2017). Mental training is also equally important and includes concentration, emotional control and mental resistance exercises (Piepiora et al., 2022a). It should be noted here that mountain climbing with the use of oxygen devices is no longer considered to be an extreme sport, but qualified mountaineering tourism (Pomfret, 2006) or active tourism (Corneliu et al., 2012). Qualified mountaineering tourism is associated with a high frequency of undertaking such expeditions while active tourism is characterized by the occasional nature of undertaking such expeditions and their hobby-like nature (Żiżka-Salamon and Gąsienica-Walczak, 2011). Recent decades have seen an increase in the popularity of high-mountain tourism, for instance commercial expeditions to the highest mountain peaks (Białecka, 2011). Regardless of the adopted forms of mountaineering, staying at high altitudes and being exposed to numerous stress factors during expeditions have a significant impact on the climbers’ physical and mental health. Positive effects on vitality, musculoskeletal and cardiorespiratory development and mental relaxation have been observed (Żurek et al., 2022). Mountaineering also has great cognitive value related to exploration and strong emotional experiences. An additional aspect is the closeness to nature and the hedonistic value, due to the participation in a given activity in high mountain terrain (Sołtysik et al., 2018). Participants’ motives for undertaking high-mountain activities refer to feelings of happiness and pleasure, positive reinforcement and character formation (Paudyal et al., 2022). The need for solitude and isolation from the outside world and overcoming one’s own limitations is also noted (Sołtysik et al., 2019). An important social aspect is the achievement of set goals and the satisfaction of the need for respect and affiliation (Skrzypińska and Atroszko, 2015). A review of a climber’s basic motives provides an individualization of his or her mental training (Chen et al., 2012).

The above-mentioned issues form the basis for research in the field of sports psychology into the personality of mountain climbers. The point of reference here is personality, which is shaped by life experiences, contacts with people who are important to a given individual, social roles played by a given person, as well as repetitive or exceptionally intense events (Allen et al., 2013). Previous studies show that the personality of mountain climbers is typical of sportspeople: low neuroticism, high extraversion, average openness to experience and agreeableness, high conscientiousness (Piepiora, 2019). It is worth noting that athletes are distinguished from non-training people by low neuroticism, higher extraversion, openness to experience, agreeableness and conscientiousness (Steca et al., 2018). Moreover, sports champions are distinguished from other athletes by one dominant factor. In the Polish sportspeople population, according to the existing research, this is even lower neuroticism (Piepiora and Piepiora, 2021); in the Australian population, this is even higher extraversion (Allen et al., 2021); in the Turkish population is an even greater openness to experience (Tok, 2011); while in the Iranian population, this is even greater conscientiousness (Mirzaei et al., 2013).

With the above in mind, it was decided to investigate the personality of Polish mountaineers. As Alpine mountaineering and Himalayan mountaineering are characterized by physical activity at different altitudes, it was considered that this may be evident in the personality of these two groups of high altitude climbers. It was assumed that there exist personality differences of Polish climbers depending on the variety of mountaineering performed. Therefore, the aim of the study was to compare the personality differences of Polish Alpine and Himalayan climbers. A hypothesis was adopted that there are significant differences between the personality of Polish Alpine and Polish Himalayan climbers. It was assumed that Himalayan climbers should be characterized by lower neuroticism and higher openness to experience and conscientiousness than Alpine climbers, because the former activity is more dangerous and technically more difficult than the latter. Consequently, an answer to a question as to possible differences existing between Polish Alpine climbers and Himalayan climbers was sought.

## Methodology

### Researched persons

The population of Polish mountaineers (*N* = 81), including 39 women and 42 men, members of the Polish Mountaineering Association, was surveyed. All respondents agreed to participate in the study and declared that they practice an extreme sport without oxygen devices. Before taking part in the survey, respondents had to specify what type of mountaineering they undertook. On this basis, the respondents were divided into Alpine climbers (*n* = 48) and Himalayan climbers (*n* = 33). Their age ranged between 20 and 49 years, their average age was 33.85 years (SD = 7.36).

### Method

The five-factor model of personality was used as a research method. It is more widely known as the ‘Big Five’ (McCrae and Costa, 1987). It consists of five traits defined on the basis of factor analysis: neuroticism, extraversion, openness to experience, agreeableness, conscientiousness (McCrae and Costa, 1997). Neuroticism is understood as susceptibility to experiencing negative emotions. It consists of: anxiety, angry hostility, depression, impulsiveness, vulnerability, self-consciousness. Extraversion, on the other hand, is a dimension relating to the quality and quantity of social interactions, activity level, energy, ability to feel positive emotions. It consists of: gregariousness, warmth, assertiveness, activity, excitement seeking, positive emotions. The individual’s tendency toward exploration, positive valuing of life experiences, tolerance toward novelty and cognitive curiosity is regarded as openness to experience. This dimension consists of: fantasy, aesthetics, feelings, actions, ideas, and values. The dimension that characterizes the attitude toward other people, the interpersonal orientation experienced in feelings, thoughts, action – is agreeableness. It consists of: trust, straightforwardness, altruism, compliance, modesty, tendermindedness. The last dimension is conscientiousness. It characterizes the degree of organization, perseverance, motivation of an individual in goal-oriented activities and describes a person’s attitude towards work. It consists of: competence, order, dutifulness, achievement striving, self-discipline, deliberation (Costa et al., 2001). The NEO-FFI Personality Questionnaire (Costa and McCrae, 2007) was used to conduct the study. It consists of 60 self-report statements. There are 12 statements for each dimension. On a five-point scale: (1) strongly disagree; (2) disagree; (3) have no opinion; (4) agree; (5) strongly agree; the intensity of each trait is measured. Each answer can be scored from 0 to 4 points. This means that a maximum of 48 points can be obtained on a given Big Five dimension. The NEO-FFI has sex-and age-dependent norms: 15–19, 20–29, 30–39, 40–49, and 50–80 years. The raw scores obtained are then converted into stens. Each Big Five trait is considered on a scale of 1 to 10 stens. Low scores are considered to be those between 1 and 3 sten. Moderate scores are between 4 and 6 sten. When scores between 7 and 10 sten are obtained, the results are interpreted as high.

### Procedure

The study was conducted between October 2021 and March 2022. The research was conducted online due to the COVID-19 pandemic restrictions in force in Poland at the time. The criterion for inclusion in the study was the voluntary willingness of the members of the Polish Mountaineering Association to participate. Subjects had 1 h to work with NEO-FFI questionnaire. The SURVIO software was used for conducting the Computer Assisted Web Interview research method. Each of the Polish mountain climbers belonging to Polish Mountaineering Association, received temporary access to the NEO-FFI questionnaire *via* e-mail, along with a request to complete it. All respondents consented to the processing of the obtained results for the scientific research purposes and read the filling instructions before commencing the questionnaire. The project was carried out following the positive opinion of the Senate Committee for Research Ethics at Wrocław University of Health and Sport Sciences number 20/2019.

### Statistical analysis

The respondents’ answers were organized using Excel spreadsheet. Statistical analyses were carried out using IBM SPSS Statistics 27.0 software. The program performed an analysis of basic descriptive statistics and a two-way ANOVA analysis of variance. The level of statistical significance in the following section was assumed at *α* = 0.05. The sensitivity analysis for the compute required effect size of the statistical test was performed, assuming the size of the research sample equal to *N* = 81, level of *α* = 0.05 and the statistical power of the test corresponding to 80% (G*power) it is possible to detect effect size ηp2 = 0.09 (Cohen’s *f* = 0.31).

## Results

As a first step, the basic descriptive statistics of the subjects were analyzed, together with the interpretation of the mean intensity of personality traits based on the Polish NEO-FFI norms. Sex differences were taken into account. Therefore, the analyses used results converted into stens according to the current Polish NEO-FFI norms (Table 1).

**Table 1:** Basic descriptive statistics for individual personality traits of mountaineers.

	*M*	Mdn	SD	Sk.	Kurt.	Min	Max	Interpretation of the average severity of the trait
Neuroticism	4.57	4.00	2.16	0.36	−0.56	1.00	10.00	Moderate
Extraversion	6.25	6.00	2.34	−0.29	−0.50	1.00	10.00	Moderate
Openness to experience	6.81	7.00	2.09	0.04	−1.07	3.00	10.00	High
Agreeableness	6.30	6.00	2.30	−0.31	−0.24	1.00	10.00	Moderate
Conscientiousness	5.56	6.00	1.98	−0.12	0.23	1.00	10.00	Moderate

M, mean; Mdn, median; SD, standard deviation; Sk., skewness; Kurt., kurtosis; Min, minimum value; Max, maximum value.

In the second phase, the assumptions of the two-factor analysis of variance were tested. An analysis of normality of distribution was performed by group of Alpine climbers and Himalayan climbers. The analysis showed that the distribution of the variables was close to a normal distribution (*p* > 0.050) with the exception of openness to experience among women in the Alpine mountaineering group, agreeableness among women in the Alpine mountaineering and Himalayan mountaineering groups and conscientiousness among men in the Himalayan climbers group (Table 2). Additionally, homogeneity of variance (*p* > 0.050) was observed across groups for all personality traits. Therefore, it was considered appropriate to use a two-factor analysis of variance.

**Table 2:** Results of the normality of distribution test for individual personality traits by group.

	Alpine climber				Himalyan climber			
	Female (*n* = 24)		Male (*n* = 24)		Female (*n* = 15)		Male (*n* = 18)	
	S-W	*p*	S-W	*p*	S-W	*p*	S-W	*p*
Neuroticism	0.95	0.250	0.94	0.079	0.93	0.270	0.90	0.056
Extraversion	0.97	0.155	0.91	0.056	0.96	0.725	0.92	0.123
Openness to experience	0.87	0.006	0.96	0.165	0.88	0.056	0.94	0.281
Agreeableness	0.91	0.043	0.92	0.176	0.85	0.019	0.95	0.460
Conscientiousness	0.94	0.699	0.88	0.041	0.92	0.209	0.88	0.030

S-W, Shapiro–Wilk normality test; *p*, significance level.

In the next step, it was tested whether Alpine mountaineers and Himalayan mountaineers differed in the intensity of individual personality traits. A two-factor analysis of variance was performed in which the independent variable was group (Alpine climbers vs. Himalayan climbers) and sex (female vs. male). Thus, both the effect of group and sex in the severity of individual personality traits were tested. Before proceeding with the analysis, the chi-square test was used to check that the groups were equal. The analysis showed that there were no statistically significant differences in the number of individuals in each group, χ^2^(1) = 0.16, *p* = 0.821. Detailed results of main effects and interactions are presented in Table 3, while the results of the pairwise comparisons test are presented in Table 4, and the confidence intervals in Table 5.

**Table 3:** Coefficients of the two-factor analysis of variance model for individual personality traits.

Model	Effect		df1	df2	*F*	*p*	η_p_^2^	η^2^
Neuroticism	Main	Sex	1	77	2.33	0.131	0.029	0.029
		Group	1	77	0.41	0.526	0.005	0.005
	Interaction	Sex * group	1	77	1.41	0.240	0.018	0.017
Extraversion	Main	Sex	1	77	0.65	0.422	0.008	0.008
		Group	1	77	1.20	0.278	0.015	0.015
	Interaction	Sex * group	1	77	0.04	0.838	0.001	<0.001
Openness to experience	Main	Sex	1	77	3.75	0.056	0.046	0.044
		Group	1	77	1.62	0.207	0.021	0.019
	Interaction	Sex * group	1	77	1.95	0.166	0.025	0.023
Agreeableness	Main	Sex	1	77	0.02	0.879	<0.001	<0.001
		Group	1	77	**5.05**	**0.027**	**0.062**	0.060
	Interaction	Sex * group	1	77	2.20	0.142	0.028	0.028
Conscientiousness	Main	Sex	1	77	3.95	0.050	0.049	0.049
		Group	1	77	0.02	0.874	<0.001	<0.001
	Interaction	Sex * group	1	77	0.16	0.689	0.002	0.002

Bold values indicate statistically significant results. df, degrees of freedom; F, ANOVA test statistic; *p*, statistical significance; η_p_^2^, partial η^2^; η^2 –^, eta square.

**Table 4:** Mean values and standard deviations with pairwise comparisons tests for simple effects.

	Alpine climber				Himalayan climber			
	Female (*n* = 24)		Male (*n* = 24)		Female (*n* = 15)		Male (*n* = 18)	
	*M*	SD	*M*	SD	*M*	SD	*M*	SD
Neuroticism	4.33	2.33	4.50	2.23	4.07	1.83	5.39	2.03
Extraversion	6.21	2.41	6.75	2.36	5.73	2.19	6.06	2.41
Openness to experience	7.21	1.84	6.96^c^	2.16	7.27^a^	2.25	5.72^ac^	1.96
Agreeableness	7.17^a^	2.37	6.33	1.99	5.27^a^	2.63	5.94	2.04
Conscientiousness	5.96	2.07	5.25	1.65	6.07	1.71	5.0	2.37

^a^Statistically significant Sidak-corrected pairwise comparison results (simple effects); ^c^ Sidak-corrected pairwise comparison results *p* = 0.056.

**Table 5:** Results of pairwise comparisons with confidence intervals.

		Neuroticism	Extraversion	Openness to experience	Agreeableness	Conscientiousness
		CI difference (95%)	CI difference (95%)	CI difference (95%)	CI difference (95%)	CI difference (95%)
Female	Male	−0.74 (−1.72–0.23)	−0.43 (−1.50–0.63)	0.90 (−0.02–1.82)	0.08 (−0.94–1.09)	0.89 (−0.01–1.78)
Alpine climber	Himalayan climber	−0.31 (−1.28–0.66)	0.58 (−0.48–1.65)	0.59 (−0.33–1.51)	**1.14 (0.13–2.16)**	0.07 (−0.82–0.96)
Female Alpine climber	Male Alpine climber	−0.17 (−1.40–1.07)	−0.54 (−1.90–0.81)	0.25 (−0.92–1.42)	0.83 (−0.46–2.12)	0.71 (−0.42–1.84)
Female Himalayan climber	Male Himalayan climber	−1.32 (−2.82–0.18)	−0.32 (−1.96–1.32)	**1.54 (0.12–2.97)**	−0.68 (−2.24–0.89)	1.07 (−0.30–2.44)
Male Alpine climber	Male Himalayan climber	−0.89 (−2.22–0.45)	0.69 (−0.77–2.16)	1.24 (−0.03–2.50)	0.39 (0.43–3.37)	0.25 (−0.97–1.47)
Female Alpine climber	Female Himalayan climber	0.27 (−1.14–1.68)	0.47 (−1.07–2.02)	−0.58 (−1.40–1.28)	**1.90 (0.43–3.37)**	−0.11 (−1.40–1.18)

Bold values indicate statistically significant results. Difference, difference of averages; CI, 95% confidence interval for the difference.

The analysis showed a significant main effect for agreeableness *F*(1.77) = 5.05; *p* = 0.027. Alpine climbers (*M* = 6.30; SD = 2.30) have higher levels of agreeableness than Himalayan climbers (*M* = 5.64; SD = 2.20). However, there was no significant effect of sex or interaction of group with sex. Interestingly, pairwise comparisons with the Sidak correction showed that, when controlling for sex, significant differences in levels of agreeableness were only observed among women. The main effect of sex for openness to experience and the main effect of sex for conscientiousness were at the limit of statistical significance. They are not described because these effects are statistically insignificant.

## Discussion

Nowadays, there is a great increase in interest in mountaineering (Krzemieniecki and Barczyński, 2019) as developments in technology have made its much more accessible (Cieśliński et al., 2016). Nevertheless, its varieties still differ in the degree of potential health and life threat (Piepiora and Piepiora, 2020; Piepiora Z. et al., 2022). Mountaineers report that they often give up other activities to undertake this form of activity (Ryn, 1969): training and climbing trips are a life priority for these individuals (Skorupa and Draga, 2012). Thus, passion and lifestyle come to the fore (Cronin, 1991). Moreover, risky mountaineering can be addictive (Paszkiewicz, 2016). It is mostly practiced in teams, therefore climbers’ social competences should be developed to a high degree (Bermúdez, 1999). Consequently, discussions on ethical issues applicable in high mountains are still a contentious issue (Vollrath and Torgersen, 2002). It remains an individual matter for modern climbers whether reaching the summit is more important than the ‘brotherhood of the rope’ and the life of another person in a critical situation (Castanier et al., 2011). It should be noted that those who undertake high altitude climbing are knowingly taking a risk to their health or life (Heska-Kwaśniewicz, 2019).

By referring the above issues to our research results, only one significant difference between the personalities of Polish Alpine climbers and Polish Himalayan climbers was noted in the study. It was in the agreeableness dimension, and only between women. Female Alpine climbers were shown to have significantly higher agreeableness than female Himalayan climbers. Female Alpine climbers have high levels of openness to experience and agreeableness and moderate levels of neuroticism, extraversion, and conscientiousness. In contrast, female Himalayan climbers have high levels of openness to experience and other Big Five dimensions at moderate levels. Alpine climbers, on the other hand, show high levels of extraversion and openness to experience and the other personality traits are at moderate levels. Interestingly, Himalayan climbers are distinguished by moderate levels of all personality traits. Discussing the obtained results, it was noted that the personality traits of Polish mountaineers are distributed differently. They are determined by the variety of climbing – Alpine or Himalayan – and the sex of those undertaking this type of competitive sport. But there is a statistically significant difference only between the levels of agreeableness of Polish female Alpine climbers and female Himalayan climbers: the former have a higher agreeableness than the latter. Thus, they are more positive towards other people. They are interpersonally oriented toward altruism being guided in their behavior by the good of others and are willing to make sacrifices. They experience this in their feelings, thoughts and actions. With regard to ethical issues in the mountains, it is reasonable to believe that female Alpine mountaineers have a greater tendency to stick with the group and provide assistance in critical situations. It has been found that the social competence of female Alpine climbers in high mountains is developed much more than that of female Himalayan climbers. And the latter, in turn, like male high-mountain climbers, are moderately agreeable. They are characterized by balanced altruism versus antagonism. From this perspective, it is reasonable to think that Polish Himalayan climbers and Polish male Alpine climbers, depending on their current priorities in the mountains, will stick together as a group and provide assistance in critical situations or they will not do so. In this sense, one can speak of hostility resulting from conflicting interests and a desire to compete. The results obtained confirm the individual issues of ethical dilemmas in high mountains (Ewert, 1994; Cooper et al., 2000; Woodman et al., 2008).

Other occurring personality differences of Polish mountaineers are not statistically significant. It is important that the personality of people practicing mountaineering is modified by the specificity of this extreme sport (Piepiora, 2021; Piepiora et al., 2022b). But the reports of other researchers (Gray, 1968; Freixanet, 1991; Clarke and Robertson, 2005; Monasterio et al., 2014; Sołtysik et al., 2019) stating that depending on the varieties of high-mountain climbing and cultural factors (nationality), the personality traits of people undertaking different varieties of high-mountain climbing are distributed differently, are also valid. It has been noted that despite these differences, in general, high-mountain climbers tend to seek adventure and risk (Self et al., 2007; Llewellyn and Sanchez, 2008; Castanier et al., 2010; Kalina, 2020). A similar trend is noted in the Far Eastern martial arts, where, in a direct confrontation between two competitors, the self-improvement of practitioners is prioritized (Litwiniuk et al., 2009, 2019). Moreover, high-mountain climbing did not lose its popularity in Poland during the COVID-19 pandemic (Klimczak et al., 2021; Fedyk et al., 2022a,b).

### Limitations of the research

The present study is limited in time and space to the surveyed Polish population of high altitude climbers. The obtained results cannot be related to the whole Polish, European or world population of high altitude climbers. They only allow us to formulate regularities relating to specific locations – the Alps and the Himalayas – and to the individual experiences of the people surveyed.

## Conclusion

There is a significant difference between the personalities of Polish Alpine climbers and Polish Himalayan climbers in the agreeableness dimension only among women. Female Alpine mountaineers are characterized by higher agreeableness than female Himalayan climbers. Himalayan climbers are otherwise not distinguished from Alpine climbers by lower neuroticism and higher openness to experience and conscientiousness. The recorded score in agreeableness was found to relate to ethical issues in the high mountains: the social competences of female Alpine climbers are considerably more developed than those of female Himalayan climbers.

## Data availability statement

The original contributions presented in the study are included in the article/supplementary material, further inquiries can be directed to the corresponding author/s.

## Ethics statement

The studies involving human participants were reviewed and approved by Senate Committee for Research Ethics at Wrocław University of Health and Sport Sciences number 20/2019. The patients/participants provided their written informed consent to participate in this study.

## Author contributions

PP and JN contributed to conception and design of the study and organized the database. PP performed the statistical analysis. PP, JB, and ZP wrote the first draft of the manuscript. PP, JB, KW, and ZP wrote sections of the manuscript. All authors contributed to manuscript revision, read, and approved the submitted version.

## Conflict of interest

The authors declare that the research was conducted in the absence of any commercial or financial relationships that could be construed as a potential conflict of interest.

## Publisher’s note

All claims expressed in this article are solely those of the authors and do not necessarily represent those of their affiliated organizations, or those of the publisher, the editors and the reviewers. Any product that may be evaluated in this article, or claim that may be made by its manufacturer, is not guaranteed or endorsed by the publisher.
